# Iatrogenic Arteriovenous Fistula Secondary to Percutaneous Coronary Intervention Causing Severe Decompensated Heart Failure

**DOI:** 10.7759/cureus.27934

**Published:** 2022-08-12

**Authors:** Nimit Kasliwal, Wilson B Pfeiffer, John F Eidt, Daniel C Gunn, Saravanan Ramamoorthy

**Affiliations:** 1 Department of Anesthesiology, Texas A&M College of Medicine, Dallas, USA; 2 Department of Anesthesiology, Brooke Army Medical Center, San Antonio, USA; 3 Department of Surgery, Baylor University Medical Center, Dallas, USA; 4 Department of Anesthesiology, Baylor University Medical Center, Dallas, USA; 5 Anesthesiology, U.S. Anesthesia Partners, Dallas, USA

**Keywords:** high svo2, severe coronary artery disease, arteriovenous fistula stent, iatrogenic arteriovenous fistula, heart failure

## Abstract

Congestive heart failure has long been a well-known cause of both morbidity and mortality for thousands of people worldwide. Consequences of decompensated heart failure are systemic and widespread, including but not limited to pulmonary edema, dyspnea, hypoxia, peripheral edema, and end-organ hypoperfusion. Common etiologies of congestive heart failure include systemic hypertension, coronary artery disease, longstanding alcohol abuse, valvular dysfunctions, and myocarditis. While the vast majority of congestive heart failure cases are secondary to one of these common etiologies, there is a subset of cases that cannot be traced to any of these causes and are most often grouped under the category of idiopathic. One rarely seen etiology of decompensated heart failure is an arteriovenous fistula, whether naturally occurring or iatrogenic. We report a case of an iatrogenic AV fistula secondary to percutaneous coronary intervention causing severe decompensated heart failure that was successfully treated with surgical ligation.

## Introduction

Arteriovenous fistulae (AVF) are most commonly encountered in the setting of renal insufficiency. Arteriovenous access via a graft or fistula is intended to be a high-flow conduit to facilitate hemodialysis. Over time, the decrease in systemic vascular resistance results in secondary increases in cardiac output, which may, in turn, result in symptomatic heart failure in susceptible patients [1]. However, AVF are also known to be congenital in origin [2-3]. In addition to intentional creation, AVF can be acquired after penetrating trauma, endovascular repair, aneurysmal rupture, and even less commonly, as a complication of diagnostic or therapeutic angiographic procedures such as cardiac catheterization [3-6]. The incidence of all forms of vascular complication following percutaneous arterial catheterization is 1% to 9%, but a prospective study of over 10,000 patients determined the incidence of developing AVF post-catheterization to be much lower, at 0.006% to 0.86% [4]. There is no described incidence of congestive heart failure resultant from iatrogenic arteriovenous fistulae. Though numerous reports exist in the literature surrounding AVF for hemodialysis, there are less data regarding iatrogenic AVF.

## Case presentation

A 62-year-old morbidly obese male with remote, right above-knee amputation was admitted with an acute exacerbation of congestive heart failure. Past medical history was notable for severe coronary artery disease status-post coronary artery bypass graft (CABG), preexisting heart failure with reduced ejection fraction (estimated 25%), and recent acute myocardial infarction status post percutaneous coronary intervention three months prior. This was complicated by a large, right groin post-procedural hematoma and the subsequent development of an AVF from the right superficial femoral and profunda femoral artery branches to the right common femoral vein. The AVF was thought to be contributing to his worsened heart failure symptoms. Percutaneous placement of a covered stent in the right superficial femoral artery was unsuccessful in controlling the AVF. Further attempts at percutaneous treatment were judged unlikely to be beneficial given the unfavorable anatomy. After a multidisciplinary discussion, the decision was made to proceed with surgical ligation of the fistula under general anesthesia. A pulmonary artery catheter was placed, which demonstrated severe pulmonary hypertension. Subsequent transesophageal echocardiography (TEE) revealed severely depressed biventricular function (left ventricular ejection fraction (LVEF) estimated at less than 10%).

Despite having had a recent myocardial infarction, multiple factors argued for urgent surgical intervention of the arteriovenous fistula. Of particular interest was his history of above-knee amputation on the ipsilateral side of the AVF. There was concern that blood flow to the remaining distal extremity would be reduced and would compromise the integrity of the stump. This has been described in the hemodialysis population as ischemic steal syndrome (ISS) or, more recently, digital hypoperfusion ischemic syndrome (DHIS) when as a result of upper extremity AVF access [7-8].

**Figure 1:**
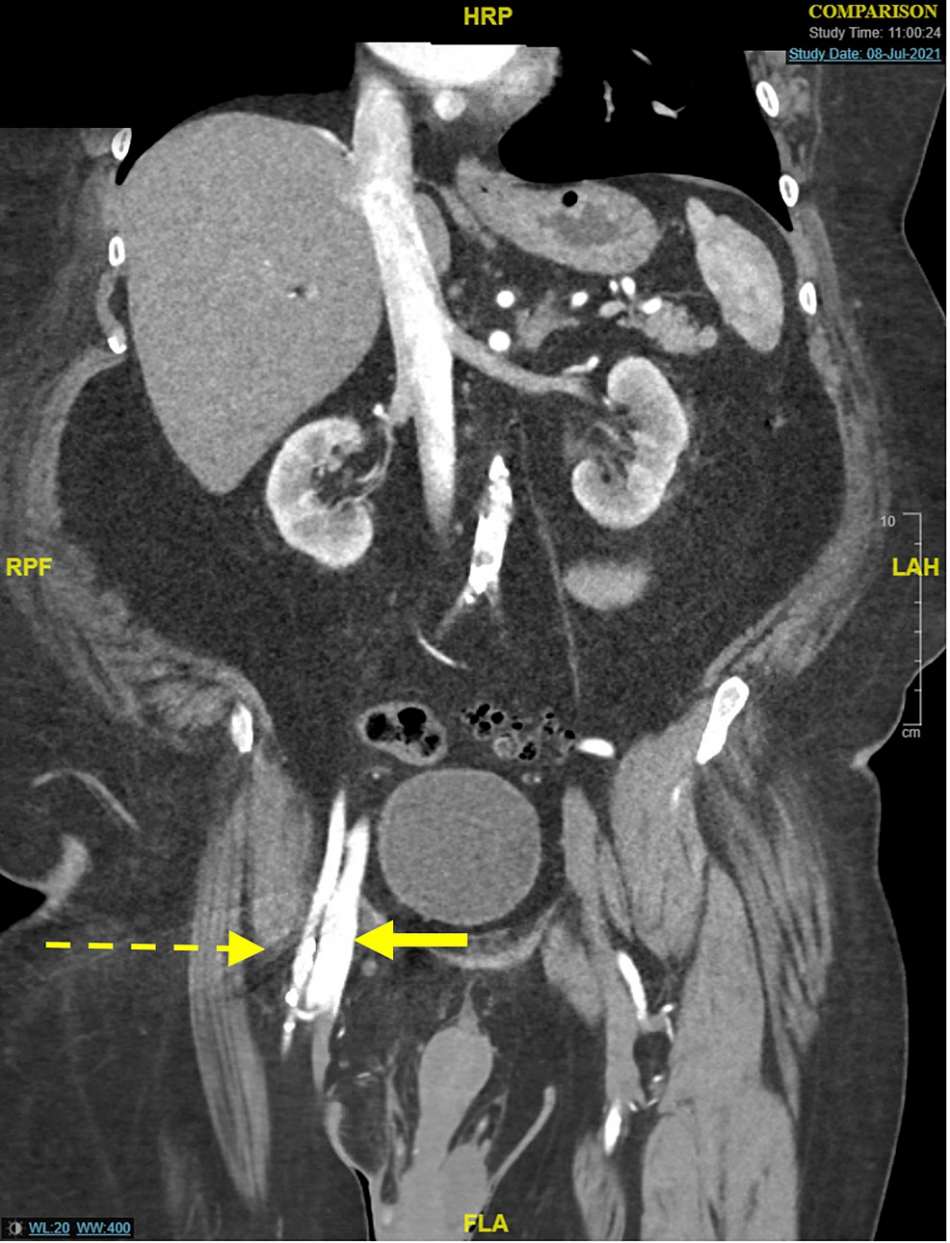
CT angiography demonstrating communication between the right superficial femoral (dashed arrow) and profunda femoral artery branches to the right common femoral vein (bolded arrow)

**Figure 2:**
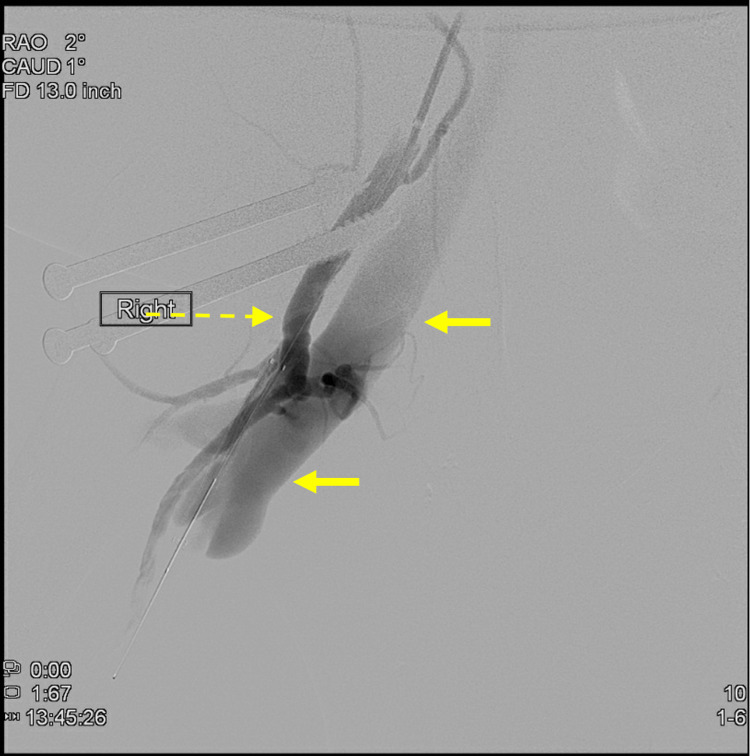
Digital subtraction angiography (DSA) of an arteriovenous fistula between the right superficial femoral (dashed arrow) and profunda femoral artery branches to the right common femoral vein (bolded arrow)

Outpatient CT angiography demonstrated a large arteriovenous fistula between the right superficial femoral and profunda femoral artery branches and the right common femoral vein (Figure 1). For this reason, he underwent proximal right superficial femoral artery stenting and angioplasty three weeks before admission; further stenting or coil embolization was not possible due to the unfavorable anatomy. Intraoperative digital subtraction angiography (DSA) demonstrated similar findings compared to his outpatient CT (Figure 2). In addition, the patient’s heart failure had been increasingly difficult to control as an outpatient. No formal echocardiography or invasive studies had been performed to evaluate the degree of decompensation. Further preoperative optimization was not feasible and would not have offered significant benefit in this case.

The patient was brought to the OR, a pre-induction radial arterial line was placed, general anesthesia was induced, and the patient was endotracheally intubated uneventfully. A pulmonary artery catheter was placed, which demonstrated a cardiac output of 7.5 L/min, an elevated CVP (29 mmHg), severe pulmonary hypertension (77/24 mmHg), and a systemic vascular resistance of 625 dynes/sec/cm^-5^, plus a mixed venous oxygen saturation of 80%. TEE revealed severely depressed biventricular function with an estimated LVEF of less than 10%. Surgical exposure of the AVF was uncomplicated. A test clamp was applied across the fistula to determine the hemodynamic contribution from fistula flow. At the clamp, heart rate decreased from 82 to 75 bpm, a decrease in stroke volume from 100 mL to 70 mL based on TEE measurements and pulmonary artery catheter measurements, a decrease in venous oxygen saturation (SvO2) from 80% to 65%, a decrease in pulmonary artery pressures from 75/26 to 60/18, a decrease in central venous pressure from 28 mmHg to 20 mmHg, and a marginal increase in systemic vascular resistance to 700 dynes/sec/cm^-5^ were noted. As the hemodynamic parameters confirmed the role of fistula flow in heart failure etiology, the decision was made to proceed with the final ligation of the AVF. Five minutes after ligation of the AVF, SvO2 of 64%, pulmonary artery pressure of 56/16 mmHg, heart rate of 74 bpm, stroke volume of 70 mL, and systemic vascular resistance of 705 dynes/sec/cm^-5^ were noted. These laboratory and hemodynamic parameters confirmed that the patient’s iatrogenic AVF was contributing significantly to his acute heart failure decompensation. After the completion of the procedure, the patient was subsequently extubated, and he was transferred to the ICU for further monitoring. The patient made an uneventful recovery with an increase in LVEF to 40% and improved RV function at the time of discharge on the fourth postoperative day.

## Discussion

Arteriovenous fistula creation is one of the most common surgical procedures done worldwide on a daily basis. While AVF creation is generally seen as a minor procedure with minimal risk involved perioperatively, it is important to recognize the risks and/or adverse events that can happen either acutely or in the future for patients undergoing AVF creation. As discussed above, one of those risks is potential decompensated congestive heart failure as a result of the AVF.

If there is concern that an AVF is contributing to physiologically significant declines in function, several assessments can be performed. It is important to recognize and understand the various monitoring modalities that can be utilized to identify whether or not an existing AVF is contributing to decompensated heart failure, and perhaps more importantly, whether ligation of the AVF will return the patient to a physiologically stable state.

First and foremost, if there is a question as to whether an AVF exists, it is important to understand the various ways of confirming its presence or absence. Arteriography can determine if a connection between the arterial and venous systems does indeed exist, although this is an invasive procedure and exposes the patient to radiocontrast material and radiation, as well as the risks of accessing the vasculature [9-11]. Doppler ultrasound is a noninvasive method that can be used to analyze the blood flow of the AVF and the shunt volume across the AVF. Graft or fistula size, patency, and overall structure can also be assessed [11-12].

After confirmation of an AVF, it is then important to assess whether or not the AVF is contributing to the decompensated physiologic state and what parameters to look for before and after ligation of the AVF. Manual compression at the bedside can be performed to indirectly assess the degree of shunt blood flow by acutely increasing systemic vascular resistance. Known as Branham’s sign, acute compression of the AVF causes a baroreceptor-mediated reflex decrease in heart rate if the shunt flow is substantial enough. There is an accompanying increase in blood pressure and a decrease in cardiac output. This maneuver was positive when used preoperatively on our patient, which aided in predicting surgical success [13-15]. Mixed venous oxygen saturation from the pulmonary artery catheter is an important parameter to note, which is expected to decrease after ligation of the AVF. After ligation of AVF, the mixed venous oxygen saturation dropped significantly, as expected. Eventually, functional improvement in heart failure due to improved systolic function is expected after ligation of the AVF. After ligation, you should expect an increase in both the right and left ventricular functions, which was seen in our case. Finally, pulmonary artery pressure is another monitor that can be utilized. After ligation of the AVF, the pulmonary artery pressure would be expected to decrease due to the reduction of arterial to venous flow. Thus, the information gathered from pulmonary artery catheterization and transesophageal echocardiography is vital when evaluating the relationship between AVF and heart failure.

## Conclusions

This case emphasizes the role of iatrogenic arteriovenous fistulae in heart failure etiology and the success of surgical ligation of arteriovenous fistulae in improving the left ventricular systolic and diastolic functions.
